# Signals vs Causation: What Observational Data Show and What They Cannot Prove

**DOI:** 10.1016/j.jacadv.2026.102600

**Published:** 2026-02-13

**Authors:** Milan Milojevic, Patrick W. Serruys

**Affiliations:** a Dedinje Cardiovascular Institute, Belgrade, Serbia; b University Hospital Zurich, University of Zurich, Zurich, Switzerland; c CORRIB Research Centre for Advanced Imaging and Core Laboratory, University of Galway, Galway, Ireland

Ren et al^1^ present registry data suggesting that total arterial revascularization (TAR) is associated with superior long-term survival across ages, including those aged ≥70 years. Several clarifications would strengthen the paper and avoid overinterpretation. First, despite statistical adjustments, residual confounding, particularly selection bias, remains likely. As shown in their Table 2,^1^ TAR recipients had fewer comorbidities, less complex disease, and more elective presentations, consistent with preferential selection of fitter patients with favorable targets. The absence of surgeon- and institution-level clustering as well as frailty assessment indices further limits the ability to adjust for these effects. Second, the registry does not capture graft patency, myocardial infarction, or repeat revascularization; thus, the biologically plausible claim that avoiding saphenous vein grafts drives the survival advantage cannot be confirmed mechanistically. Third, heterogeneity in techniques, periprocedural complications (eg, deep sternal wound infection, radial-artery vasospasm), and learning-curve effects caution against broad generalization.^2^

Furthermore, the discussion failed to include the findings from the SYNTAX Extended Survival study, both the coronary artery bypass graft (CABG)-only analyses (single vs multiple vs total arterial grafts) and the percutaneous coronary intervention-versus-CABG stratified by conduit configuration. In the CABG cohort, at 12.6 years, mortality was 34.6% with non-TAR vs 19.1% with TAR,^3^ and survival improved stepwise with greater arterial coverage (lowest with percutaneous coronary intervention; then 1- and 2-territory arterial CABG; highest with 3 territories).^4^ Citing these data would triangulate and contextualize the The Australian and New Zealand Society of Cardiac and Thoracic Surgeons (ANZSCTS) findings, thereby strengthening external validity.

Moreover, the paper stratifies patients by age (≥70 vs <70 years) and reports separate models, but it does not present a primary overall effect for TAR vs non-TAR across the whole cohort. Methodological standards require reporting the overall treatment effect first, then testing a formal treatment-by-age interaction, with subgroup estimates labeled as exploratory and multiplicity addressed.

Finally, a registry-cohort mismatch also warrants clarification. The present study analyses primary isolated CABG with ≥2 grafts, 2001 to 2020 (n = 59,641), whereas a prior paper from the same group reports 54,275 patients receiving ≥2 grafts in “primary not isolated” operations.^5^ Reconciling definitions and denominators would aid interpretation.
